# Isolation of Extracellular Vesicles From Microalgae: A Renewable and Scalable Bioprocess

**DOI:** 10.3389/fbioe.2022.836747

**Published:** 2022-03-14

**Authors:** Angela Paterna, Estella Rao, Giorgia Adamo, Samuele Raccosta, Sabrina Picciotto, Daniele Romancino, Rosina Noto, Nicolas Touzet, Antonella Bongiovanni, Mauro Manno

**Affiliations:** ^1^ Cell-Tech Hub, Institute of Biophysics, National Research Council of Italy, Palermo, Italy; ^2^ Cell-Tech Hub, Institute for Research and Biomedical Innovation, National Research Council of Italy, Palermo, Italy; ^3^ Department of Biological, Chemical and Pharmaceutical Sciences and Technologies, University of Palermo, Palermo, Italy; ^4^ Centre for Environmental Research Innovation and Sustainability, Institute of Technology Sligo, Sligo, Ireland

**Keywords:** nanoalgosomes, extracellular vesicles, renewable biosources, microalgae, tangential flow filtration

## Abstract

Extracellular vesicles (EVs) play a crucial role as potent signal transducers among cells, with the potential to operate cross-species and cross-kingdom communication. Nanoalgosomes are a subtype of EVs recently identified and isolated from microalgae. Microalgae represent a natural bioresource with the capacity to produce several secondary metabolites with a broad range of biological activities and commercial applications. The present study highlights the upstream and downstream processes required for the scalable production of nanoalgosomes from cultures of the marine microalgae *Tetraselmis chuii*. Different technical parameters, protocols, and conditions were assessed to improve EVs isolation by tangential flow filtration (TFF), aiming to enhance sample purity and yield. The optimization of the overall bioprocess was enhanced by quality control checks operated through robust biophysical and biochemical characterizations. Further, we showed the possibility of recycling by TFF microalgae cells post-EVs isolation for multiple EV production cycles. The present results highlight the potential of nanoalgosome production as a scalable, cost-effective bioprocess suitable for diverse scientific and industrial exploitations.

## 1 Introduction

Extracellular vesicles (EVs) are a diverse group of membranous nanoparticles originated from cells and involved in several biological processes (Yáñez-Mó et al., 2015; Margolis and Sadovsky, 2019). EVs perform specific and selective cargo release to cells or target tissues via different mechanisms, including endocytosis, fusion, or receptor interaction, and in general they take part in intercellular signal transduction (Van Niel et al., 2018; Raposo and Stahl, 2019; Limongi et al., 2021a). Beyond their physiological functions, EVs have a role in several diseases, including cancer (Vagner et al., 2018; Raimondi et al., 2020), and in numerous pathological conditions, for instance, in stimulating an immune response (Zhou et al., 2020) or intervening in multidrug resistance in cancer treatments (Samuel et al., 2017; Pasini and Ulivi, 2020) and in virus infections and transmission (Urbanelli et al., 2019; Pocsfalvi et al., 2020). Due to their intrinsic capability to vehicle biological materials and information, EVs have high potential as drug delivery systems (Armstrong and Stevens, 2018; Kooijmans et al., 2021). Indeed, there is an increasing interest to exploit EVs as therapeutics (Witwer et al., 2019; Silva et al., 2021; Limongi et al., 2021b) and in a large variety of biotechnological applications (Paganini et al., 2019).

A growing interest is also arising from the study and exploitation of EVs or, more in general, of micro- and nano-sized vesicles, derived from non-human sources, such as bacteria (Bitto and Kaparakis-Liaskos, 2017), bovine milk (Kleinjan et al., 2021; Samuel et al., 2021), and edible plants (Wang et al., 2014; Raimondo et al., 2015; Bokka et al., 2020; Stanly et al., 2020; Raimondo et al., 2021). In particular, plant-derived vesicles are currently considered as biocompatible, sustainable, green, next-generation nanocarriers (Kameli et al., 2021; Urzì et al., 2021).

In such a context, we recently identified microalgae as a novel natural source of EVs, called nanoalgosomes or simply algosomes (Adamo et al., 2021; Picciotto et al., 2021). Microalgae are microorganisms (mostly photosynthetic and autotrophic) considered a promising source of natural bioactive macromolecules such as pigments, polyunsaturated fatty acids, vitamins, and polysaccharides (Cuellar-Bermudez et al., 2015; Khan et al., 2018). These compounds are well known to possess a wide range of biological functions including antioxidant, antibacterial, antiviral, and anticancer activities. Microalgae operate the biosynthesis of numerous health beneficial compounds; thus, they are exploitable as natural ingredients in functional foods or cosmetics, with a corresponding attention from industries (Caporgno and Mathys, 2018; Khan et al., 2018; Jacob-Lopes et al., 2019; Morocho-Jácome et al., 2020).

In our previous studies, we evaluated different microalgal species for their capability to produce EVs (Picciotto et al., 2021) and we identified the marine chlorophyte microalga *Tetraselmis chuii (T. chuii)* as one of the most promising biosource for a large-scale production of EVs (Adamo et al., 2021). Beyond its capability to produce EVs with a high yield, *T. chuii* has also an interesting content of valuable natural pigments, including lutein and *β*-carotene (Picciotto et al., 2021), and it has been approved as a novel food for human consumption (European Commission, 2017). Nanoalgosomes exhibit remarkable benefits in comparison with EVs derived from other sources, such as mammalian cells, plant, bacteria, or milk, since microalgae are a non-animal, sustainable biosource and also a fast-growing organism that can be easily cultured in large scale under controlled conditions (Adamo et al., 2021).

In the present work, we focus on the isolation of nanoalgosomes and we optimize an efficient bioprocess for a sustainable, scalable, and renewable EVs production, along with a robust quality control procedure, as defined in our previous work (Adamo et al., 2021) in accord to the guidelines and the consensus from the scientific community (Théry et al., 2018). A cost-effective and reliable EVs production, which is also suitable for an industrial or large scale exploitation, requires a fine tuning of both upstream and downstream processes (Paganini et al., 2019; Buschmann et al., 2021; Grangier et al., 2021; Staubach et al., 2021). Here, we discuss and define a clear manufacturing practice for the implementation of nanoalgosome production, with optimized protocols for microalgal cultivation (***upstream processing***) and isolation of EVs by Tangential Flow Filtration (TFF), an isolation technique allowing to process large volumes of microalgae cultures, reaching concentrated EV samples (***downstream processing***). Our production pipeline is optimized thanks to quality controls, ensured by an extensive biophysical and biochemical characterization by different techniques, including dynamic light scattering (DLS), nanoparticle tracking analysis (NTA), immunoblot analysis (IB analysis) of protein markers, atomic force microscopy (AFM), and BicinChoninic Acid assay (BCA assay) (Romancino et al., 2018; Adamo et al., 2021; Paganini et al., 2021). Moreover, we demonstrate the possibility to recycle microalgal biomass after EVs harvesting to renew the cell culture and continue EVs production in a cyclic bioprocess (***renewable processing***). This capability to go through several production/isolation cycles further increases the interest of microalgae as a sustainable and renewable biosources of EVs.

## 2 Materials and methods

### 2.1 Microalgae cultivation

A stock culture of the microalgae *Tetraselmis chuii (T. chuii)* (CCAP 66/21b) was grown in borosilicate glass bottles in modified f/2 medium (Guillard, 1975) and used to start new cultures in bottles via a 25% v/v inoculum. Cultures were kept for 4 weeks at a temperature of 22°C ± 2°C under continuous air flow and exposed to white light with a photoperiod of 14 h light and 10 h dark. Bottles were gently shaken every 2 days in order to homogenize cultures. Microalgae were cultured in sterile conditions by using 0.22 *μ*m filters at the bottle inlets. The cell growth was monitored every week by optical density at 600 nm, and cell counting (see Supplementary Figure S1).

### 2.2 Tangential flow filtration

The KrosFlo^®^ KR2i TFF System from Repligen (Spectrum Labs, Los Angeles, CA, USA) was used to isolate microalgae-derived EVs. Microalgae cultures (1.6 L) were clarified by sequential micro- and ultra-filtration using TFF hollow fiber filters (MiniKros Sampler) with cut-off of 650 nm (S04-E65U-07-N, Spectrum Labs), 200 nm (S04-P20-10-N, Spectrum Labs), and 500-kDa (S04-E500-10-N, Spectrum Labs). Three different settings were evaluated: feed flow 750 ml/min and permeate flow 60 ml/min, feed flow 450 ml/min and permeate flow 6 ml/min, and feed flow 450 ml/min and permeate flow 6 ml/min followed by a wash of the TFF cartridges with 100 ml of culture medium. During all filtration processes transmembrane pressure (TMP) was kept constant at 0.02 bar. The small and large EVs recovered from the retentate of the 500-kDa and 200 nm cut-off TFF filter modules, respectively, were concentrated until a final volume of almost 150 ml. Subsequently, using a smaller 500-kDa cut-off TFF filter module (C02-E500-10-N, Spectrum Labs, MicroKros) with a feed flow 75 ml/min and a permeate flow 2 ml/min, samples were further concentrated and diafiltrated seven times with PBS, reaching a final volume of approximately 5 ml.

### 2.3 Microalgae cultivation recycling protocol

After culture clarification, the retentate obtained from the 650 nm cut-off TFF cartridge (100 ml) was diluted in modified f/2 medium to reach the initial batch volume (1.6 L) and used to start renewed cultures in bottles via a 25% v/v inoculum. After 4 weeks of cultivation, microalgae were again processed by TFF to isolate EVs. In order to maintain sterile conditions, necessary for the recycle of the microalgae cell culture, the 650 nm TFF membrane filter was washed with 1 L of sterile water before starting the clarification process. Moreover, for the first TFF step the instrument and the bioreactors are connected in a closed system to maintain sterility.

### 2.4 Nanoparticle tracking analysis

Measurement of nanoparticle size distribution and concentration was performed using NanoSight NS300 (Malvern Panalytical, United Kingdom). The NTA instrument is composed of a 488 nm laser, a high sensitivity sCMOS camera, and a syringe pump. In order to achieve the suggested concentration measurement range (10^7^ ÷ 10^8^ particles per ml) in which 20 ÷ 120 particles per frame were tracked, the EVs-enriched samples have been diluted in particle-free water. The analysis of the samples was executed using the NanoSight Software NTA 3.4 Build 3.4.003 (camera level 15–16, syringe pump speed 30) acquiring five videos of 60s duration and examining 1,500 frames for each sample. The frame analysis was carried out setting a detection threshold so that the observed particles are marked (red crosses in the software) and no more than five particles are rejected (blue crosses). Medium viscosity was set to water viscosity. As in our previous work (Adamo et al., 2021), nanoalgosomes may be equivalently diluted both in water and in PBS since ionic strength has no effect on their integrity (see supporting information, Supplementary Figure S2).

### 2.5 Protein content (BCA assay)

The EVs protein content was quantified using the colorimetric BCA protein assay (Thermo Fisher Scientific, Rockford, IL, USA). The protein concentration was measured at 562 nm, according to the manufacturer’s instructions, using a GloMax^®^ Discover Microplate Reader.

### 2.6 Immunoblotting

The Western blot analysis was executed using sodium dodecyl-sulfate (SDS) polyacrylamide gel electrophoresis (PAGE); 10 *μ*g of cell lysate and 5 *μ*g EV samples (in PBS) were incubated at 100°C for 5 min with 5× loading buffer (0.25 M Tris-Cl pH 6.8, 10% SDS, 50% glycerol, 0.25 M dithiothreitol, 0.25% bromophenol blue) and loaded on 10% SDS polyacrylamide gel for electrophoresis. Polyvinylidene fluoride (PVDF) membranes are used to blot proteins. The membranes were blocked with BSA-TBS-T solution [3% powdered with bovine serum albumin in TBST (50 mM Tris HCl pH 8.0, 150 mM NaCl, 0.05% Tween 20)] for 1 h at room temperature, followed by primary antibody incubation. The antibody anti-Alix (clone 3A9, dil. 1:150 in 3% BSA/TBS-T1X), incubated overnight at 4°C, is raised against a mammalian EV marker and is cross-reactive for microalgae. The antibody anti-H+/ATPase (dil. 1:1,000 in 3% BSA/TBS-T1x, Agrisera), incubated for 1 h at room temperature, is raised against H+/ATPase a membrane protein specific for plants and protists. After washing, membranes were incubated for 1 h with secondary antibodies according to the manufacturer’s instructions (horseradish peroxidase-conjugated secondary anti-mouse or anti-rabbit antibodies, cell signaling), and then washed four times in TBST for a total of 20 min. Immunoblots were revealed using SuperSignal™, Pierce™ ECL (Thermo Fisher Scientific).

### 2.7 Dynamic light scattering

An aliquot of vesicle solution was pipetted and centrifuged at 1,000×*g* for 10 min at 4°C in order to remove any dust particles. The supernatant was withdrawn by pipet tips (previously washed by MilliQ water), put directly into a quartz cuvette and incubated at 20°C in a thermostated cell compartment of a BI200-SM goniometer (Brookhaven Instruments) equipped with a He-Ne laser (JDS Uniphase 1136P) with wavelength *λ* = 633 nm and a single pixel photon counting module (Hamamatsu C11202-050). Scattered light intensity and its autocorrelation function *g*_2_(*t*) were measured simultaneously at a scattering angle *ϑ* = 90° by using a BI-9000 correlator (Brookhaven Instruments). Absolute scattered intensity, namely excess Rayleigh ratio, *R*_*ex*_, was obtained by normalization with respect to toluene: $R_{ex}=\left[I-I_{B}\right]\cdot I_{T}^{-1}\cdot {\tilde{n}}^{2}\cdot {\tilde{n}}_{T}^{-2}\cdot R_{T}$ , where I, I_*B*_, and I_*T*_ are the scattered intensities of sample, buffer, and toluene, respectively; $\tilde{n}$ = 1.336 7 and ${\tilde{n}}_{T}$ = 1.499 6 are the refractive indexes of buffer and toluene at 633 nm, respectively; and *R*_*T*_ is the toluene Rayleigh ratio at 633 nm (R_*T*_ = 14 × 10^−6^ cm^−1^) (Noto et al., 2012). The intensity autocorrelation function *g*_2_(*t*) is related to the size *σ* of diffusing particles and to their size distribution *P*_*q*_(*σ*), by the relation $g_{2}\left(t\right)=1+{\left|\beta \int P_{q}\left(\sigma \right)\exp\left[-D\left(\sigma \right)q^{2}t\right]\right|}^{2}$ , where *β* is an instrumental parameter, $q=4\pi \tilde{n}\lambda^{-1}sin\left[\vartheta /2\right]$ is the scattering vector, and *D*(*σ*) is the diffusion coefficient of a particle of hydrodynamic diameter *D*_*h*_ = *σ*, determined by the Stokes-Einstein relation *D*(*σ*) = *k*_*B*_ *T* [3*πησ*]^−1^, with *T* being the temperature, *η* the medium viscosity, and *k*_*B*_ the Boltzmann constant (Berne and Pecora, 1990). The size distribution *P*_*q*_(*σ*) is calculated by assuming that the diffusion coefficient distribution is shaped as a Schultz distribution, which is a two-parameter asymmetric distribution, determined by the average diffusion coefficient $\bar{D}$ and its variance $\bar{v}$ (Berne and Pecora, 1990; Romancino et al., 2018). This approach is justified by the typical noise level in the autocorrelation functions (Mailer et al., 2015). Two robust parameters may be derived from this analysis: *D*_*z*_, the z-averaged hydrodynamic diameter (the diameter corresponding to the average diffusion coefficient $\bar{D}$ ), and *PDI*, the polydispersity index $\left(PDI=\bar{v}{\bar{D}}^{-2}\right)$ , which is an estimate of the distribution width. The integrity of nanoalgosomes has been shown by measuring DLS autocorrelation function in different hypo and hyper tonic solutions from 0 to 300 mM NaCl. The same EVs sample has been dialyzed against the different solutions for 2 h at room temperature and, after dialysis buffers change, overnight at 4°C. No effect is observable in both size distribution and particle number (see supporting information, Supplementary Figure S2).

### 2.8 Atomic force microscopy

A 40*μ*l vesicle solution, diluted in MilliQ water to a final concentration of a few *μ*g/ml, was deposited onto freshly cleaved mica, incubated for 20 min, and gently dried under nitrogen flow. Tapping mode AFM measurements were carried out by using a Nanowizard III scanning probe microscope (JPK Instruments AG, Germany) equipped with a 15 *μm* z-range scanner and NSC-15 (Mikromasch) cantilevers (spring constant 40 N/m, typical tip radius 8 nm); 2 × 2 *μ*m^2^ images were acquired at 256 × 256 pixel resolution. Setpoint was fixed at 70% of free oscillation amplitude (20 nm). Other measurements were performed in liquid by quantitative imaging upon deposition on a functionalized substrate (see supporting information, Supplementary Figure S3).

## 3 Results and discussion

### 3.1 Upstream processing

**FIGURE 1:**
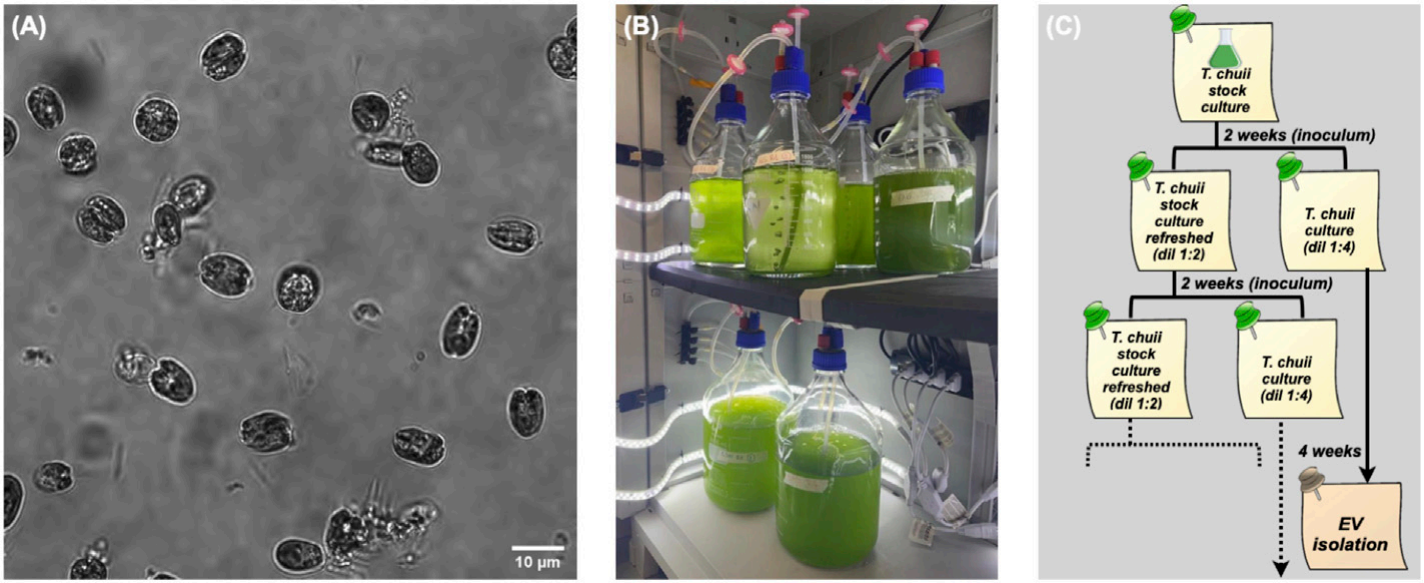
Microalgal cultivation. **(A)** Microscopy image (60×) of *T. Chuii* cells. **(B)** Lab cabinet with parallel bioreactors. **(C)** Flow chart of the cultivation timing.

In the present work, we isolated EVs from the marine chlorophyte microalgae *T. chuii* (Figure 1A). This species was selected from a set of several microalgal strains as one of the best candidates for EVs production (Adamo et al., 2021; Picciotto et al., 2021). We established a permanent platform for microalgae cultivation at pilot scale.

Different cultivation methods are available for microalgae, in brief (Henley, 2019):

• ***batch***—a given volume of culture medium is inoculated with cell culture at low density and then processed during exponential growth to obtain a maximum yield;
• ***fed batch***—a supplement of culture medium and nutrients periodically feeds the cell culture without removing the biomass before harvesting;
• ***semicontinuous***—a fixed volume of cell culture is harvested at given time intervals and replaced with fresh culture medium;
• ***continuous***—the harvested culture is drained out of and the fresh medium is fluxed in the bioreactor continuously to maintain a constant biomass concentration.

Although a continuous method may in principle allow a higher yield, we preferred to implement on a lab/pilot scale a batch cultivation in small bioreactors (a few liters each), since it is reliable and it facilitates the setting of sterile conditions. Both the method and the apparatus are well suitable for scaling-out. Thus, we typically cultivate several liters of cultures, which are synchronously inoculated, grown, and distributed in different bioreactors, as shown in Figure 1B. The harvested cultures are then pooled and processed at the same time.

The harvesting time depends upon the life cycle of *T. chuii* as well as the culture conditions, such as the amount of the inoculated culture. Specifically, after 4 weeks the cell density increases to reach a maximum, as measured by periodic optical density measurements and cell counting. Along with the cultures fated to EV production, we maintained a refreshed stock culture, as described in the general flow chart of Figure 1C.

### 3.2 Downstream processing

#### 3.2.1 Isolation by differential tangential flow filtration

Differential ultra-centrifugation (dUC) is the classical methods for EV isolation and purification (Théry et al., 2006). It consists of a series of subsequent centrifugation and eventually ultracentrifugation steps to progressively remove and fractionate cells, debris, large particles, and small particles. While its protocols are well established (Théry et al., 2018), dUC is not easily suitable for large-scale EV production, since it is time-consuming and low throughput, due to the various centrifugation steps (Coumans et al., 2017). Also, it presents further drawbacks, including EV aggregation (Linares et al., 2015; Yuana et al., 2015); coisolation of contaminants, e.g. protein aggregates (György et al., 2011; Paolini et al., 2016); and damage of EV structure due to high shear forces (Ismail et al., 2013; Coumans et al., 2017). Other isolation methods have been used for viruses or virus-like particles and then exploited for EV isolation, due to their close structural analogy (Merten et al., 2016). These methods include density gradient ultracentrifugation (gUC), filtration, and various chromatographies (Staubach et al., 2021), such as size exclusion chromatography (SEC), ion exchange chromatography (IEX), and affinity chromatography (AC) (Paganini et al., 2019).

A reliable method extensively used for liposomes (Worsham et al., 2019) as well as for virus isolation (Loewe et al., 2019) and now adopted in the EV field is tangential flow filtration (TFF) (Heinemann et al., 2014; Busatto et al., 2018; Haraszti et al., 2018). In TFF, the particle solution, or the cell culture, flows tangentially over a membrane with a given size cut-off. The feed solution is circulated with low pressure by a peristaltic pump in a closed loop through the reservoir and the filter unit (which it typically a hollow fiber). A part of the solution permeates the filter and is then recovered with a content of particle with a size smaller than the pore size (permeate). At the same time, the feed volume is reduced and depleted of small particles (retentate). The same process may be used for diafiltration or effective volume reduction of the retentate, which is extremely important for subsequent use of EV products, e.g. for therapeutic application (Witwer et al., 2019). With respect to dead-end filtration, TFF considerably reduces membrane fouling and the formation of the undesirable filter cake due to the crowding of small-size particles. With respect to dUC, TFF induces a low shear stress, thus providing more gentle processing and resulting in high yield (Busatto et al., 2018; Haraszti et al., 2018). In general, TFF allows the processing of large volumes in a short time with high reliability and reproducibility. The process is then easily scalable and suitable for the production of GMP-compliant products (Bari et al., 2018).

**FIGURE 2:**
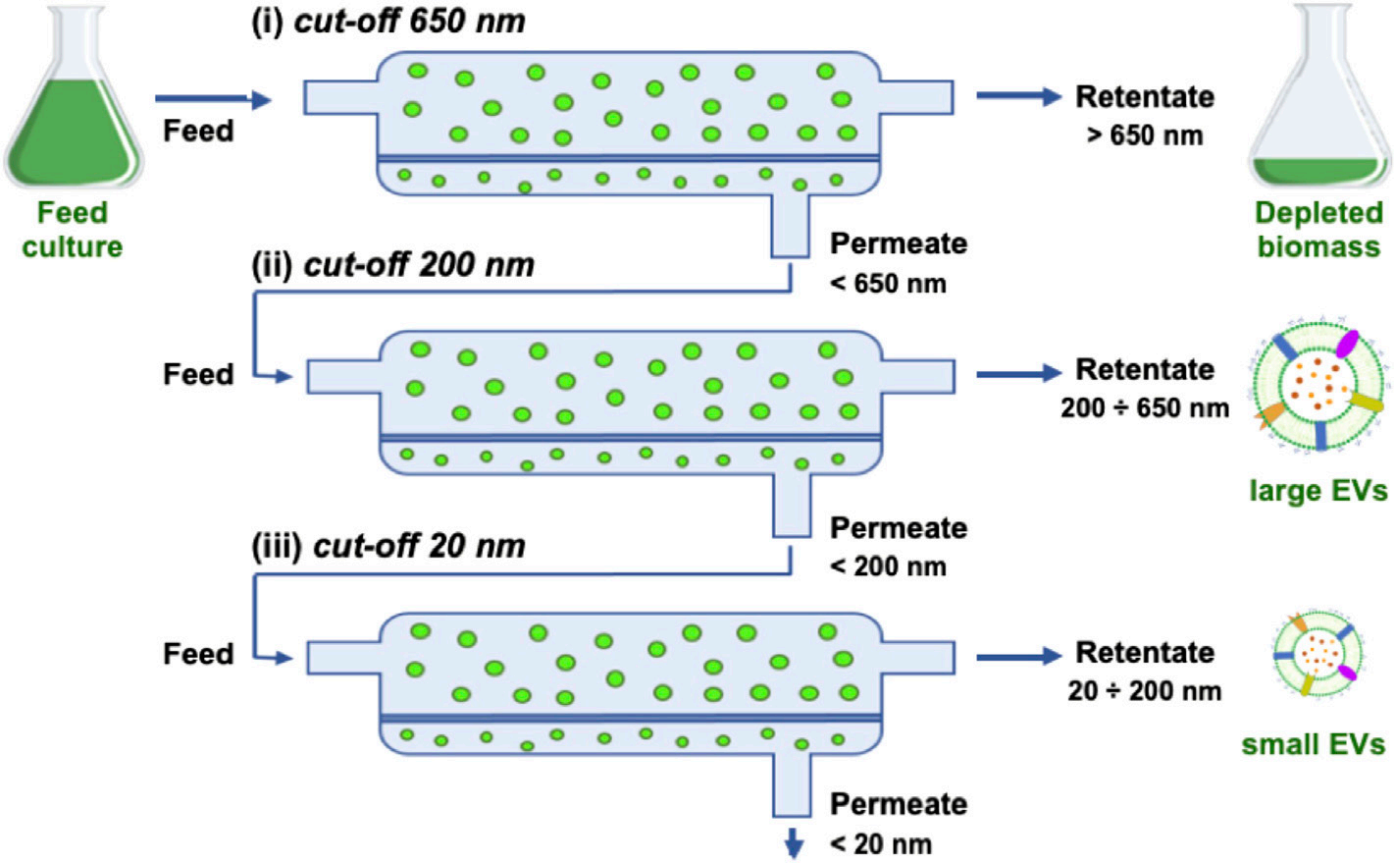
Scheme of TFF steps showing the retentate, permeate, and feed for the three filters used in sequence: (i) 650 nm, (ii) 200 nm, (iii) 20 nm (namely 500 kDa).

In our previous work, we have used both differential ultracentrifugation (dUC) and tangential flow filtration (TFF) to isolate EVs from microalgae, confirming the above described expectations for TFF performance (Adamo et al., 2021). Thus, we implemented the following procedure based on sequential TFF filtration steps (Figure 2).

1) Clarification

The harvested cell culture is fluxed through a fiber with a cut-off of 650 nm to remove the biomass.

2) Isolation

The clarified permeate from the previous step is fluxed through a fiber with a cut-off of 200 nm; this step allows to isolate EVs smaller than 200 nm in the permeate by removing larger objects, including large EVs or cellular debris which escaped the first clarification.

3) Ultrafiltration and volume reduction

The isolated permeate from the previous step is fluxed through a fiber with a cut-off of 500 kDa, which corresponds to approximately 20 nm; this step allows to remove small particles, such as proteins, maintaining small EVs in the retentate; at the same time, the retentate volume is reduced down to the fiber volume (150 ml in the current setting).

4) Concentration and diafiltration

The ultrafiltered retentate from the previous step is fluxed through a fiber with the same cut-off (500 kDa) and a lower volume and filter surface; this step allows to further concentrate the sample by reducing the volume down to the volume of the filter module and the tubing (5 ml in the current setting).

The isolation of nanoalgosomes actually occurs in step 2 after filtration via a 200 nm filter. At this stage, the retentate mainly consists of a subpopulation of large particles at very low concentration as well as a fraction of small EVs that were not brought in the permeate. The subsequent TFF ultrafiltration steps (3 and 4) are quite important to achieve a rapid volume reduction. Moreover, they are very efficient in removing any small particle, such as freely diffusing proteins. In the case of *T. chuii* culture, this purification step is made easier by the simplicity of the microalgal culture medium. At the opposite, EV purification from a complex culture medium, such as for instance in the case of mammalian cells, may require a further purification step to remove small particles (typically size exclusion chromatography), as reported in other studies and according to our own experience (Staubach et al., 2021). Indeed, a culture with high protein content may result in quick membrane fouling affecting all TFF steps and preventing an efficient recovery.

#### 3.2.2 TFF parameters optimization

In order to optimize the TFF protocol, the specific parameters controlling the process must be adjusted individually for each type of culture medium (Moleirinho et al., 2019). Two important parameters to maximize EV yield are the inlet and outlet flow rates: more specifically, the feed flow rate, F_*in*_, and the permeate flow rate, F_*out*_. We evaluated three different conditions for each filtration step:

(A) F_*in*_ = 750 ml min^−1^, F_*out*_ = 60 ml min^−1^;
(B) F_*in*_ = 450 ml min^−1^, F_*out*_ = 6 ml min^−1^;
(C) F_*in*_ = 450 ml min^−1^, F_*out*_ = 6 ml min^−1^ and filter module wash.

**FIGURE 3:**
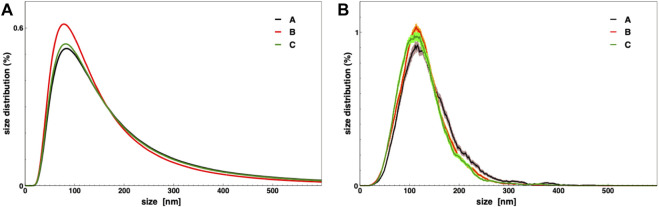
Nanoalgosomes size distribution in three TFF conditions as described in the text, measured by DLS **(A)** and NTA **(B)**.

The condition (C) adds to condition (B) the eventual wash of each cartridge with a 100 ml culture medium to avoid EVs losses on the filter membrane. In order to compare the different TFF conditions, a 1.6 L microalgae culture was portioned in three equal volume samples (≈530 ml), which were then processed by TFF. The EV-enriched samples (5 ml) resulting from the retentate of the last small column (step 4), underwent several biophysical and biochemical analyses aiming to quantify small EV isolation yields. EVs yields, for each small EVs sample obtained by TFF, were evaluated in term of protein content using the BCA assay and in terms of particles number calculated by NTA (both normalized per mg of dry microalgal biomass). Additionally, their average size and size distributions were determined by DLS and NTA (Figure 3 and Table 1). The biophysical characterization allows a straightforward evaluation of the EV yield in the chosen conditions. Figure 3 and Table 1 show that the size distribution, along with the average size, are identical in the three preparations. Other parameters are related to the amount of isolated particles, namely, the particle number measured by NTA, the total protein mass measured by BCA assay, and the excess Rayleigh ratio, corresponding to the absolute value of scattered intensity and proportional to particle concentration. Therefore, Table 1 shows that a slow flow rate determines a higher EV recovery (conditions B and C), likely due to the prevention of any membrane fouling. An additional washing step (condition C) may also improve particle recovery.

**TABLE 1:** Characterization of nanoalgosomes obtained from the three different TFF conditions (A, B, and C). D_*z*_: z-averaged hydrodynamic diameter; PDI: polydispersity index; R_*ex*_/biomass: excess Rayleigh ratio, measured by DLS, over dry biomass; N_*P*_ (*NTA*): particle number, measured by NTA, over dry biomass; c_*p*_ (*BCA*): Protein mass, measured by BCA assay, over dry biomass.

TFF	D_*z*_	PDI	R_*ex*_/biomass	N_*P*_ (*NTA*)	c_*p*_ (*BCA*)
Conditions	(nm)		(10^–6^ cm^−1^ g^−1^L)	(10^9^ g^−1^)	(*μ*g g^−1^)
A	85 ± 5	0.5	1.05 ± 0.01	320 ± 25	50 ± 7
B	85 ± 5	0.5	1.90 ± 0.01	760 ± 10	29 ± 7
C	85 ± 5	0.5	2.90 ± 0.01	810 ± 45	42 ± 4

### 3.3 Renewable processing

After the optimization of upstream and downstream processing, we were able to produce EVs at a lab scale, with a maximum yield of 2 mg EVs for every 5 L of cell culture and approximately every 5 g of dry biomass. Also, our production platform showed high reproducibility and quality over different production cycles, as discussed in the present work and in our previous studies (Adamo et al., 2021; Picciotto et al., 2021). Now, we explore the possibility to link upstream and downstream processes by implementing a cyclic bioprocess for nanoalgosomes isolation. Since microalgae cells are concentrated during the first TFF filtration step, the corresponding retentate could be used to seed a new culture via appropriate dilution with fresh medium (modified f/2 medium) and subjected to further sequential filtration after 4 weeks. This requires that microalgal cells are not damaged during the first TFF step, when the cell culture is depleted from the released vesicles.

**FIGURE 4:**
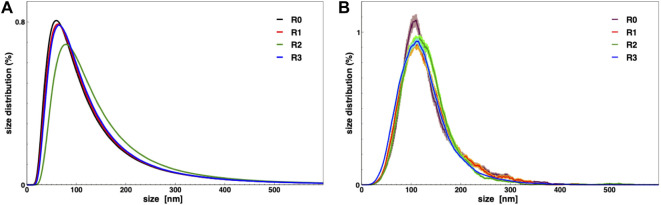
Nanoalgosomes size distribution from a fresh culture (R0) and after 1, 2, and 3 recycling (R1, R2 and R3, respectively), measured by DLS **(A)** and NTA **(B)**.

**FIGURE 5:**
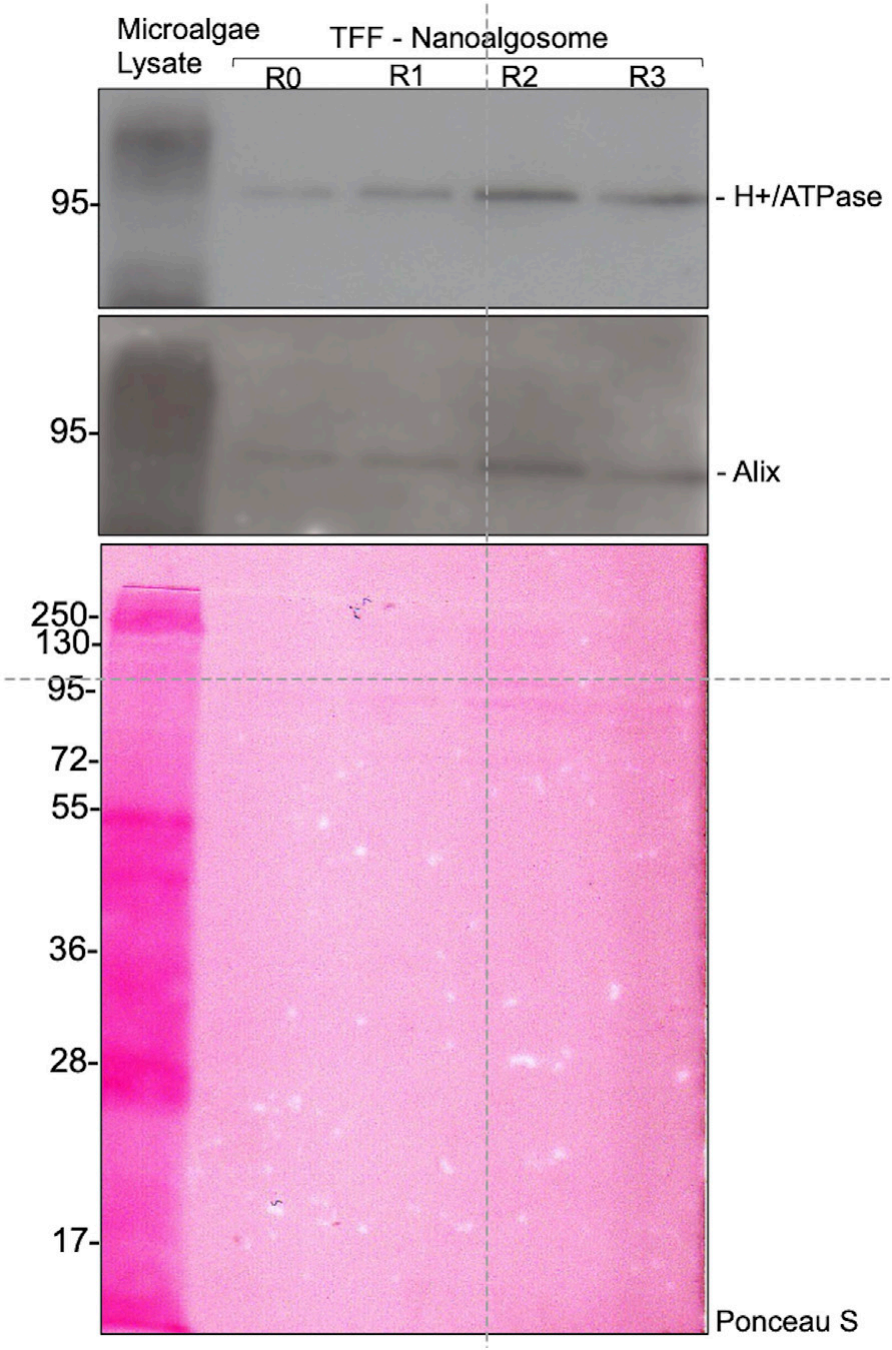
Immunoblot analysis of specific biomarkers (H+/ATPase and Alix) in *Tetraselmis chuii* cells lysate (Microalgae lysate; 10 µg) and nanoalgosomes isolated by TFF from *Tetraselmis chuii* fresh culture (R0), and after 1, 2, and 3 subculturing steps (R1, R2, and R3, respectively) (upper panel). Ponceau red staining is shown as loading control (bottom panel). Three independent experiments (n = 3) were performed.

In order to demonstrate the capability of such a production for high throughput EVs production, we characterized the nanoalgosomes obtained from a fresh culture and its subsequent TFF-based subculture cycles (up to 3). BCA assay and NTA were performed to compare and quantify the EV sample yields (Table 2). Furthermore, the average size and size distributions of the small vesicles were measured by DLS and NTA (Figure 4). Finally, the biochemical characterization was completed by assessing the presence of EVs in each sample by immunoblot analysis, emphasizing the expression of particular biomarkers (e.g., H+/ATPase and Alix) in accord with the MISEV 2018 guidelines (Théry et al., 2018) and our previous work (Adamo et al., 2021). Immunoblot results showed the enrichment of specific biomarkers (H+/ATPase and Alix) in Nanoalgosomes samples Figure 5. Specifically, semiquantitative densitometric analysis of immunoblotting showed an increase in the expression of target proteins in nanoalgosomes isolated from renewals of the *T. chuii* cultures. As a negative control, we perform immunoblot analysis to verify the absence of the biomarker TET8, which is an orthologue of mammalian tetraspanins in plants and bacteria, that is not present in *T. chuii* and here used as a negative control for the presence of bacterial contaminants. In addition to the enrichment of biomarkers, we can also observe a slight increase of EV amount over subsequent recycling, in terms of protein mass, measured by BCA assay and of total particle number determined by NTA (Table 2).

**TABLE 2:** Characterization of nanoalgosomes obtained from a fresh culture (R0) and after 1, 2 and 3 recycling (R1, R2, and R3, respectively). D_*z*_: z-averaged hydrodynamic diameter; PDI: polydispersity index; R_*ex*_/biomass: excess Rayleigh ratio, measured by DLS, over dry biomass; N_*P*_ (*NTA*): Particle number, measured by NTA, over dry biomass; c_*p*_ (*BCA*): Protein mass, measured by BCA assay, over dry biomass.

Samples	D_*z*_	PDI	R_*ex*_/biomass	N_*P*_ (*NTA*)	c_*p*_ (*BCA*)
Recycling	(nm)		(10^–6^ cm^−1^ g^−1^L)	(10^9^ g^−1^)	(*μ*g g^−1^)
R0	85 ± 5	0.50	21.00 ± 0.01	5,500 ± 300	375 ± 4
R1	85 ± 5	0.55	17.70 ± 0.01	5,500 ± 700	383 ± 8
R2	100 ± 5	0.60	19.10 ± 0.01	5,400 ± 300	425 ± 7
R3	90 ± 5	0.45	15.55 ± 0.02	11 ,400 ± 500	435 ± 4

**FIGURE 6:**
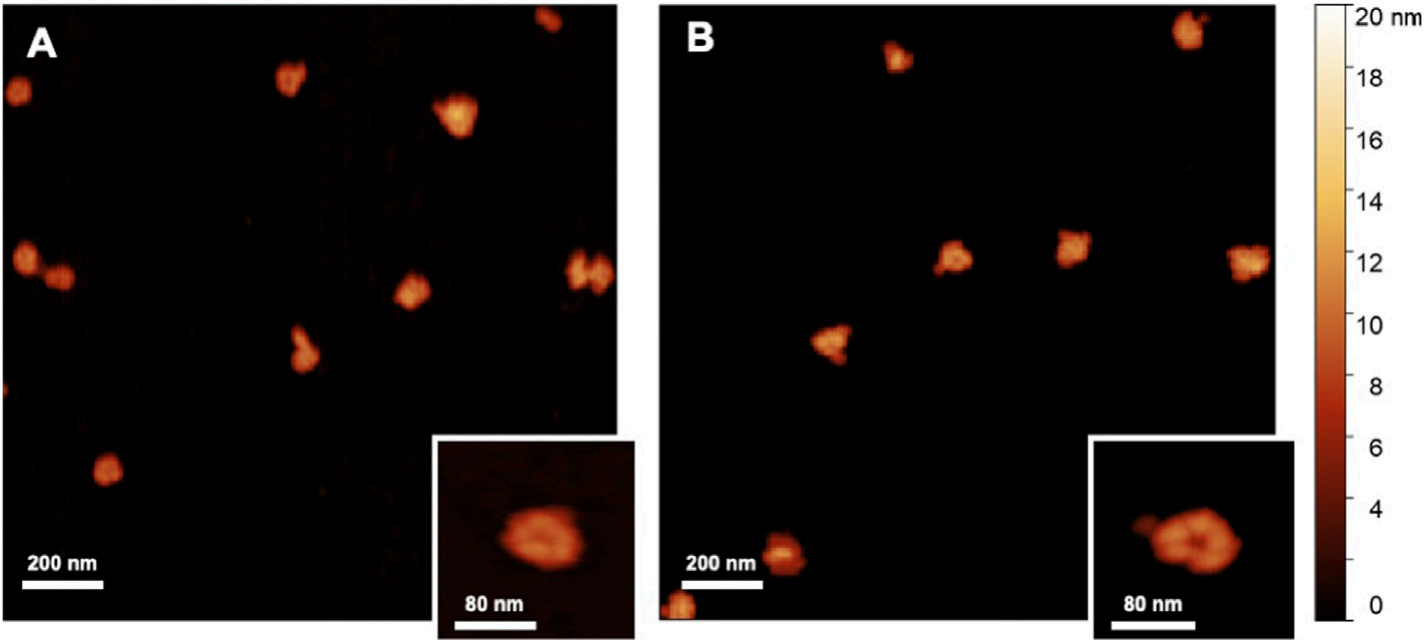
AFM images (2 × 2 *μ*m^2^) of nanoalgosomes **(A)** from a fresh culture and **(B)** from a culture after multiple recycles.

The overall population of nanolgosomes was not altered by TFF-based recycling, as shown by the unchanged size distributions, measured by DLS and NTA (Figure 4). Also the morphology of nanoalgosome was not altered, as clearly shown in the AFM images of Figure 6. Other AFM images were taken by using a functionalized substrate for amine groups, and no significant changes were observed after TFF-based subcultivation cycles (see supporting information, Supplementary Figure S3). In some cases, we observed that the recycled samples displayed a wider concentration of particles, namely, sample impurities. This may warrant further purification steps, for instance by size exclusion chromatography. The functional behavior of nanoalgosomes, which is currently under study, is not addressed in the present work. Nevertheless, given the growth of cells in culture after each TFF-based recycling regime (see supporting information, Supplementary Figure S1), it is possible that their functional properties would not be altered.

### 3.4 Hard numbers for a fast quality check

We reported different quantities related to the amount of vesicles in solution:

1) ***c***_***p***_, protein mass concentration determined by BCA assay

This quantity is a standard parameter in the EV field; its measure can be done by using a specific kit and a spectrophotometer or a colorimeter; thus it is quite cheap and easily accessible in every laboratory. Also, it is a widely used procedure and thus it is very useful to compare measurements from different studies and different samples. Other colorimetric methods measuring protein mass concentration, such as the popular Bradford assay, may be equivalently performed. On the other hand, an accurate measurement of protein concentration typically requires a concentrated sample, so it is often taken after a concentration step, which introduces the possibility of sample loss and a biased measurement.

2) ***N***_***P***_, particle number concentration measured by NTA

The number of particles is the ideal quantification for each sample and it is becoming another standard parameter with the increasing availability of NTA instruments, or also Resistive Pulse Sensing techniques, which allow to track and count each particle in a sample. There are two main drawbacks: the first is the intrinsic limit of detection of NTA instruments which are less sensitive to objects with a size below 100 nm (or with a very large size), giving a constitutive bias to the measure of number concentration; the second is the intrinsic incomplete sampling of the particle population; indeed, both a short experimental duration and a different setting of the acquisition parameters may lead to large differences in the particle count, and hence in the particle concentration, which only a highly trained operator can reliably and partly suppress.

3) ***R***_***ex***_, excess Rayleigh ratio measured by DLS

This quantity is an absolute measure of the intensity scattered at a given angle. While the measure requires an appropriate instrumentation (not every light scattering commercial device is adequate), it is very easy and requires a very low sample amount, if taken at 90°. Most importantly, since DLS intrinsically performs an exhaustive ergodic sampling of all the particles in solution, the measure is quite robust, carries an almost irrelevant error, and is not biased by any instrumental parameter or analytic method. For such a reason, it is a reliable quantity suited for the comparison of different samples or different batches. On the other hand, its physical meaning is not straightforward. It is proportional to the total mass concentration *c* of the particles in solution. However, it also proportional to the weight average mass of the particles *M_w_* and to their z-averaged form factor *P*_*z*_(*q*), which is related to the average shape of the particles and depends upon the scattering angle *ϑ*, or the scattering vector *q*: *R*_*ex*_(*q*) ∼ *cM*_*w*_ *P*_*z*_(*q*) (Berne and Pecora, 1990).

**FIGURE 7:**
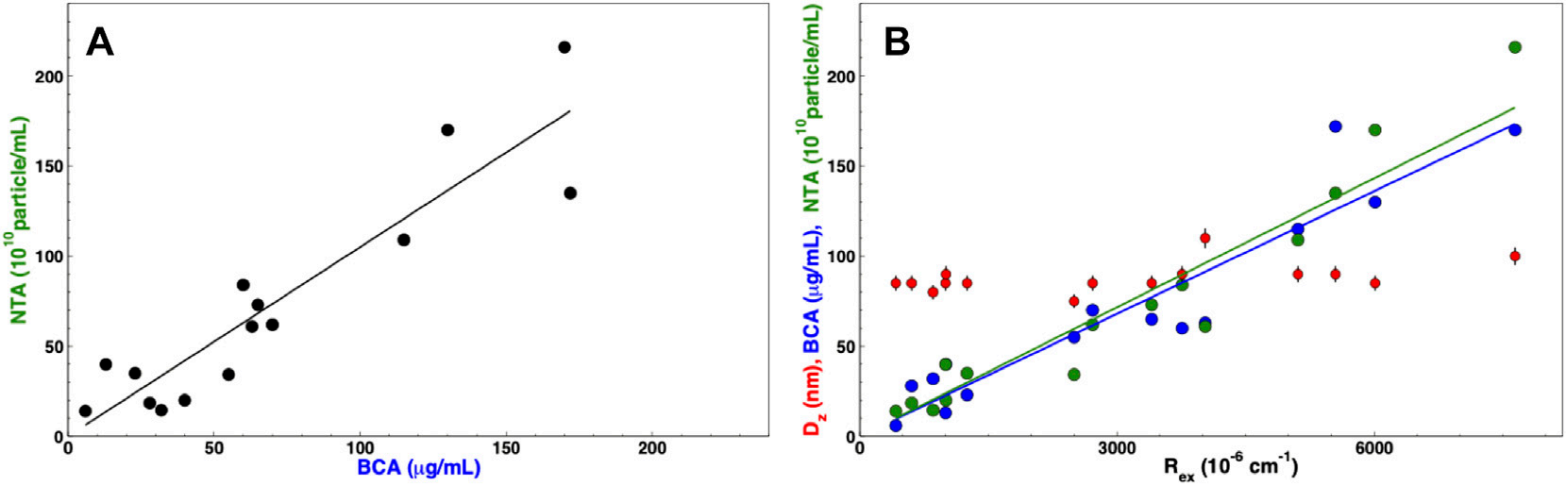
Correlation among concentration parameters: **(A)** particle concentration measured by NTA (black circles) vs. protein concentration measured by BCA assay. **(B)** Protein concentration measured by BCA assay (blue circles), particle concentration measured by NTA (green circles), and z-averaged hydrodynamic diameter (red circles) vs. excess Rayleigh ratio, R_*ex*_; The solid lines show a linear regression to data for BCA vs. R_*ex*_ (blue line) and NTA vs. R_*ex*_ (green line).

After several iterations of different purifications of nanoalgosomes with different yield but comparable quality, we are able to put some order in the information derived from these quantities. First, we observed an expected correlation between the protein concentration *c*_*p*_ and the particle number *N*_*P*_, as shown in Figure 7A: *N*_*P*_ = *Sc*_*p*_, where *S* = 10.5 × 10^9^ *μg* ^−1^, which is slightly higher than the constant calculated by Sverdlov (Sverdlov, 2012). If the correlation is evident, one may note that the variability in the data does not allow to infer one quantity by simply measuring the other one. For this reason, we recommend to measure both quantities to complete any batch characterization.

Furthermore, Figure 7B shows that both quantities are correlated with *R*_*ex*_. This result is not trivial in the case of particles with a heterogeneous size distribution. One may argue that the two quantities, the weight average mass *M_w_* (proportional to *D* ^2^), and the z-averaged form factor *P*_*z*_(*q*) (roughly proportional to *D* ^−2^ at high q) average out, thus making *R*_*ex*_ directly proportional to the mass concentration *c* (Montis et al., 2017). However this could be assumed for large particles and at large angle (i.e. in back scattering). Otherwise, in order to unravel the relation between scattered intensity and particle concentration, a more complex multi-angle analysis would be required. In our case, the strict correlation between particle number and Rayleigh ratio is likely due to the reproducible size distribution of our preparations (as observable in the quite constant average size of Figure 7B).

In any case, the correlation shown in Figure 7B works as an *a posteriori* calibration of the particle number with respect to the “hard number” of Rayleigh ratio, which can be quickly measured to assess EV concentration and make a reliable and accurate batch to batch comparison. For instance, a 90° excess Rayleigh ratio of 42 × 10^–6^ *cm* ^−1^ corresponds to a number concentration of 10^10^ particles *mL* ^−1^.

## 4 Conclusion

The role of EVs in cell communication is attracting increasing interest from several clinical and biological fields. This emerging relevance is also supported by the application of EVs for clinical diagnosis and liquid biopsy (Ayers et al., 2019; Trino et al., 2021). Also, their potential exploitation as efficient drug delivery systems boosted the interest in their biotechnological exploitation. In order to fulfill the increasing demand for EVs, it is required to adopt new strategies for their massive production at high purity level or, at least, with a controlled batch reproducibility.

Here, we addressed the production of nanoalgosomes, EVs derived from microalgae recently identified and characterized in our recent work (Adamo et al., 2021; Picciotto et al., 2021). Both upstream and downstream processing steps have been optimized to maximize EV yields. Moreover, we showed that it is possible to operate microalgal production cycles using TFF-derived culture inocula to facilitate a cyclical production of nanoalgosomes. The optimization of EV production was achieved by implementing quality control checks, which included the use of several biophysical and biochemical methods for EV characterization.

**FIGURE 8:**
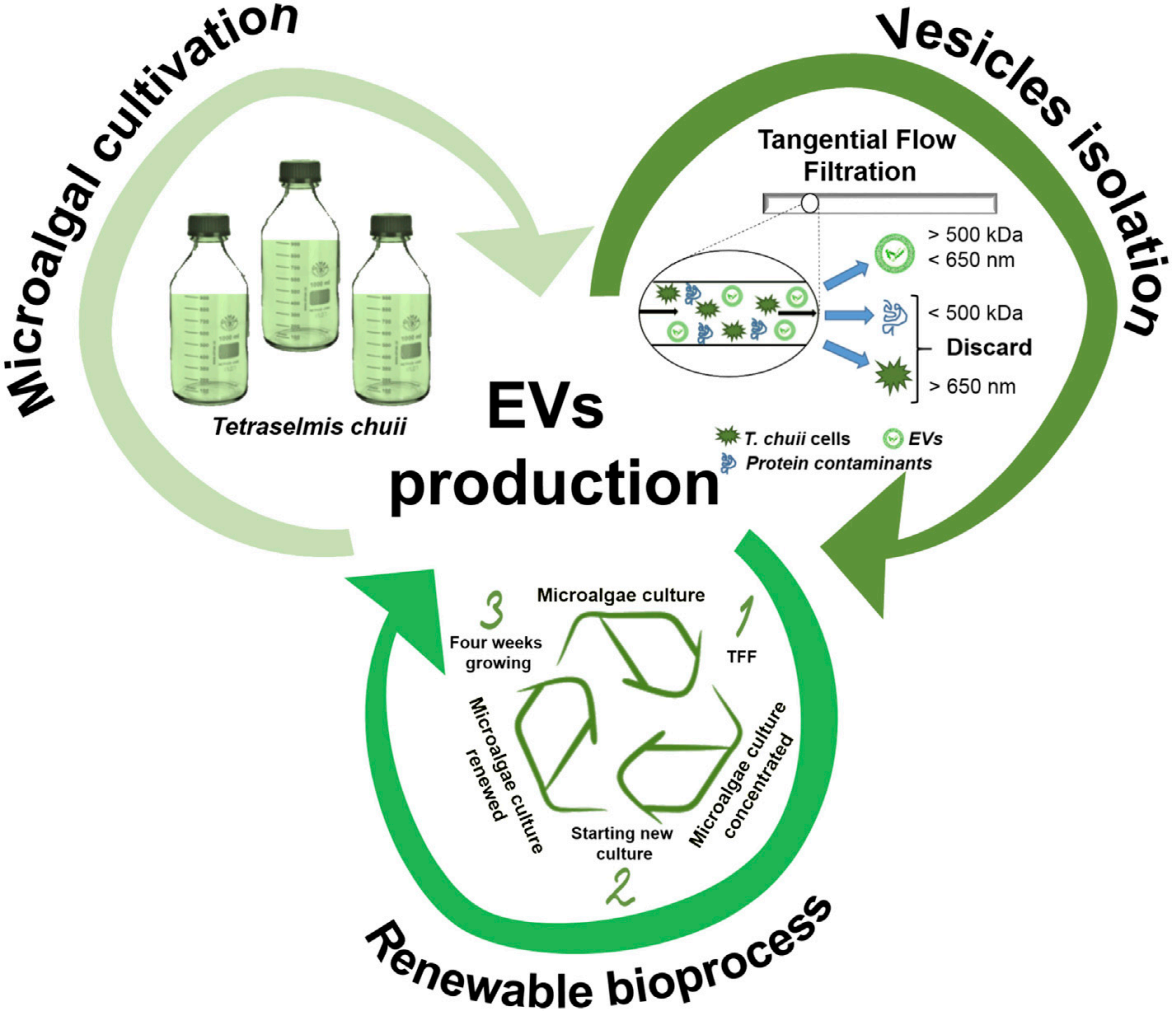
Schematic representation of EV production as a sustainable (cultivation of microalgae), scalable (isolation by TFF), and renewable bioprocess.

As highlighted in the present work and accounted in previous studies (Paganini et al., 2019; Adamo et al., 2021), nanoalgosomes have different competitive advantages with respect to EVs derived from other sources (Figure 8): 1) *Sustainability*. They are obtained by a sustainable “green” biosource: they can be seen as more appealing for an exploitation as drug carriers than EVs from human or animal sources, which have inherent safety and ethical issues. 2) *Scalability*. The optimized TFF based bioprocess is suitable for a large scale production: for any large-scale exploitation, a cell suspension has a definitive advantage with respect to other green sources, such as higher plants, which require more time-consuming and expensive treatments. 3) *Renewability*—the potential recycling of TFF-concentrated microalgal cells to facilitate the scaled production.

## Data Availability

The raw data supporting the conclusion of this article will be made available by the authors, without undue reservation.
