# Supplementary material for: Isolation of Extracellular Vesicles From Microalgae: A Renewable and Scalable Bioprocess
Source: Front Bioeng Biotechnol. 2022 Mar 14;10:836747. doi: 10.3389/fbioe.2022.836747 (PMC8963918; doi:10.3389/fbioe.2022.836747)
Supplement: Supplementary file 1 [file DataSheet1.PDF]

Supplementary Material

1 GROWTH OF MICROALGAL CELLS AFTER RECYCLING

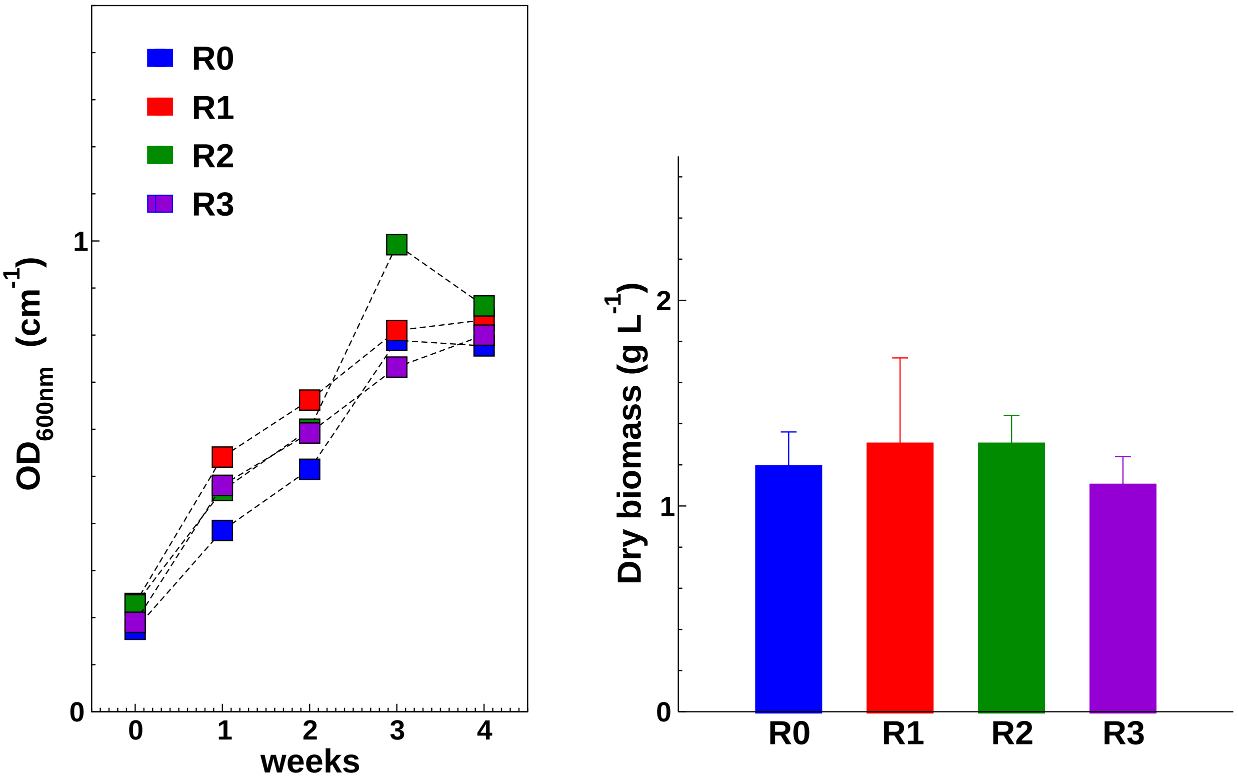

Figure S1.
Microalgal cells growth and biomass for fresh culture (R0) and after 1,2 and 3 recycling (R1, R2 and R3, respectively).
Left panel: growth of microalgal cell culture monitored by optical density (OD).
Right panel: dry biomass per liter of culture before EVs isolation (after 4 weeks of culture).

2 NO EFFECT OF IONIC STRENGTH

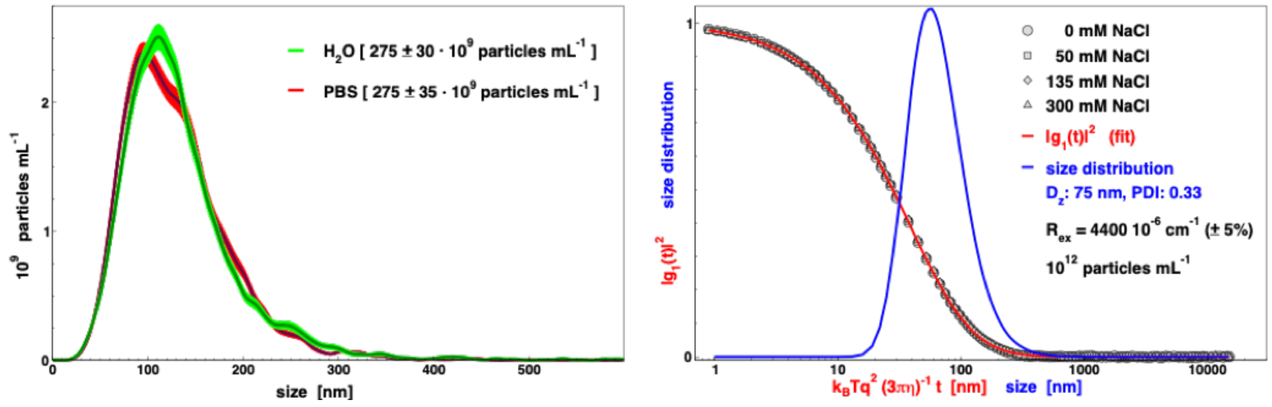

Figure S2.
Left panel: EVs size distribution measured by NTA; the same EVs solution has been diluted 1000 times in both MilliQ water and filtered PBS solution; no effects are observable in both size distribution and particle number.
Right panel: Autocorrelation functions $|g_1(t)|^2$ (grey symbols), measured by DLS, of EVs in solution with different ionic strengths (as in the figure legend). The same EVs solution has been dialysed against different solutions for 2 hours at room temperature and, after dialysis buffers change, has been dialyzed overnight at 4°C. All the correlation functions have been fitted (red curve) with a Shultz distribution resulting in a size distribution (blue curve) with z-averaged hydrodynamic diameter D_z of 75 nm and a polydispersity index PDI of 0.33. In all the samples the excess Rayleigh ratio R_{ex} is 4400 10^6 cm^{-1}, within 5%, corresponding to about 10^{12} particle mL^{-1}. No effect is observable in both size distribution and particle number.

3 FURTHER AFM IMAGES

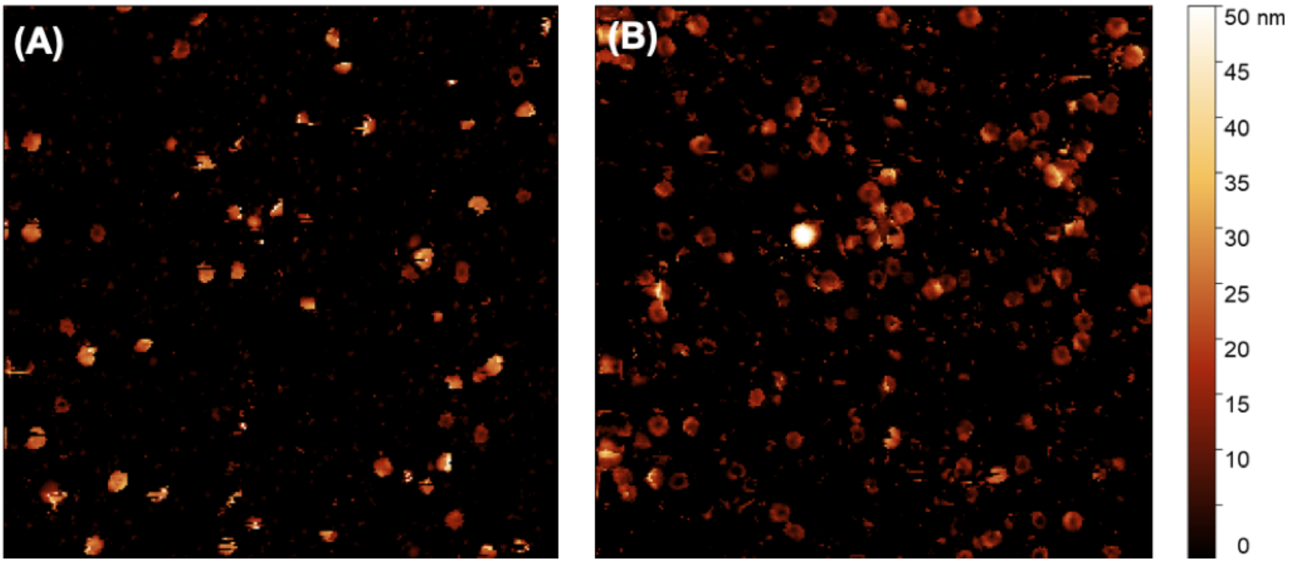

Figure S3.
AFM images (2x2 μm^2) of nanoalgosomes (A) from a fresh culture and (B) from a culture after multiple recycle.

Sample preparation: 40 ul vesicle solution, diluted in PBS to a final concentration of a few μg/mL, were deposited into APTES/glutaraldehyde functionalized glass slides and incubated for 24h; then, the sample was gently rinsed by PBS to remove non-adsorbed vesicles.
Vesicle imaging: Quantitative Imaging AFM measurements were carried out in PBS by using a Nanowizard III scanning probe microscope (JPK Instruments AG, Germany) equipped with a 15-um scanner, and AC40 (Bruker) silicon cantilevers (spring constant 0.1 N/m, typical tip radius 8 nm); 2 x 2 μm^2 images were acquired at 256x256 pixels resolution (force setpoint: 75 pN, z-length: 50 nm, pixel time 5 ms); the cantilever was thermally calibrated by using the tool in JPK software [Hutter, J. L. and Bechhoefer, J. (1993). Calibration of atomic-force microscope tips. Review of Scientific Instruments 64, 1868–1873, doi:10.1063/1.1143970].
Substrate functionalisation: (i) borosilicate glass slides were cleaned by immersion in boiling acetone for a few minutes and dried in a stream of high-purity nitrogen and exposed to UV radiation in order to expose the hydroxyl groups of silica; (ii) then they were treated with 0.25 M (3-aminopropyl)-triethoxysilane (APTES) in chloroform for 3 minutes at room temperature, and then rinsed thoroughly with chloroform and dried with nitrogen; (iii) eventually they were treated with 0.4 M glutaraldehyde aqueous solution for 3 minutes at room temperature and then rinsed with Milli-Q water and dried with nitrogen.
